# C-Type Natriuretic Peptide Induces Anti-contractile Effect Dependent on Nitric Oxide, Oxidative Stress, and NPR-B Activation in Sepsis

**DOI:** 10.3389/fphys.2016.00226

**Published:** 2016-06-23

**Authors:** Laena Pernomian, Alejandro F. Prado, Bruno R. Silva, Aline Azevedo, Lucas C. Pinheiro, José E. Tanus-Santos, Lusiane M. Bendhack

**Affiliations:** ^1^Department of Pharmacology, School of Medicine of Ribeirão Preto (FMRP), University of São PauloRibeirão Preto, Brazil; ^2^Department of Physics and Chemistry, Faculty of Pharmaceutical Sciences of Ribeirão Preto, University of São PauloRibeirão Preto, Brazil; ^3^Department of Biomechanics, School of Medicine of Ribeirão Preto (FMRP), Medicine and Rehabilitation of the Locomotor System, University of São PauloRibeirão Preto, Brazil

**Keywords:** C-type natriuretic peptide, sepsis, natriuretic peptide receptor B, reactive oxygen species, nitric oxide, phenylephrine-induced contraction

## Abstract

**Aims:** To evaluate the role of nitric oxide, reactive oxygen species (ROS), and natriuretic peptide receptor-B activation in C-type natriuretic peptide-anti-contractile effect on Phenylephrine-induced contraction in aorta isolated from septic rats.

**Methods and Results:** Cecal ligation and puncture (CLP) surgery was used to induce sepsis in male rats. Vascular reactivity was conducted in rat aorta and resistance mesenteric artery (RMA). Measurement of survival rate, mean arterial pressure (MAP), plasma nitric oxide, specific protein expression, and localization were evaluated. Septic rats had a survival rate about 37% at 4 h after the surgery, and these rats presented hypotension compared to control-operated (Sham) rats. Phenylephrine-induced contraction was decreased in sepsis. C-type natriuretic peptide (CNP) induced anti-contractile effect in aortas. Plasma nitric oxide was increased in sepsis. Nitric oxide-synthase but not natriuretic peptide receptor-B expression was increased in septic rat aortas. C-type natriuretic peptide-anti-contractile effect was dependent on nitric oxide-synthase, ROS, and natriuretic peptide receptor-B activation. Natriuretic peptide receptor-C, protein kinase-Cα mRNA, and basal nicotinamide adenine dinucleotide phosphate (NADPH)-dependent ROS production were lower in septic rats. Phenylephrine and CNP enhanced ROS production. However, stimulated ROS production was low in sepsis.

**Conclusion:** CNP induced anti-contractile effect on Phenylephrine contraction in aortas from Sham and septic rats that was dependent on nitric oxide-synthase, ROS, and natriuretic peptide receptor-B activation.

## Introduction

One of the most important clinical characteristics of sepsis and septic shock is the vascular hyporesponsivity to contractile agonists (Donaldson and Myers, 1996; Vromen et al., 1996; Strunk et al., 2001). It represents an important condition for patient survival. Different therapeutic strategies aimed to improve vital organ function (Leone and Martin, 2008). The identification of new intracellular signaling related to sepsis progression might contribute to the development of therapeutic strategies to reduce sepsis-associated mortality.

Sepsis cardiovascular dysfunction involves excessive nitric oxide (NO) production (Strunk et al., 2001) by NO-synthases (NOS) (Thomas et al., 2008). The endothelial NOS (NOS3) plays main role on organ blood flow distribution and is related to microvascular permeability regulation and cell interaction. Sand et al. (2015) showed that mesenteric blood flow decreases in a time-dependent manner and in parallel with the development of metabolic acidosis and organ dysfunction after induction of sepsis. Inducible NOS (NOS2) is constitutively expressed on few quantities and its protein expression is enhanced after inflammatory stimuli in different cells (Strunk et al., 2001). Sustained NO production following NOS2 induction is associated with sepsis hyporesponsivity (Donaldson and Myers, 1996; Vromen et al., 1996; Strunk et al., 2001). Furthermore, the role of neuronal NOS (or NOS1) seems to contribute to the hyporesponsivity of contractile adrenergic agonists in sepsis (Nardi et al., 2014). The inhibition of soluble guanylyl cyclase (sGC), enzyme which is the main target to NO, is able to recover mean arterial pressure (MAP) and enhance cardiac contractility in septic patients (Fernandes et al., 2009).

The oxidative stress is associated with impaired vasoconstriction in sepsis (Szabó et al., 1995; Salvemini and Cuzzocrea, 2002; Wu et al., 2004). According to Wu et al. (2004), antioxidant treatment, before cecal ligation and puncture (CLP) surgery to induce sepsis, increases mice survival, and decreases hypotension, plasma NO metabolites, oxidative stress, NOS2 mRNA, and angiotensin II (AngII) hyporesponsivity. Regarding to endothelial cells, Li et al. (2016) showed that lipopolysaccharide (LPS) treatment of human umbilical vein endothelial cells (HUVEC) increases oxidative stress, malondialdehyde levels, superoxide dismutase 2 (SOD2) protein expression and phosphorylation of c-Jun N-terminal kinases (JNK), and decreases SOD1 expression. Moreover, Zhou et al. (2012) observed that intravenous bolus injection of ascorbate, 30 min prior to CLP surgery or 3 h after CLP surgery, attenuated NOS activity and prevented vascular permeability increases.

The C-type natriuretic peptide (CNP) is a member of the natriuretic peptide family with high conserved amino acid sequence (Inoue et al., 2003). It can be synthesized, stored, and released from endothelial cells. CNP is present in LPS-, cytokines-, and growth factors-stimulated endothelial cells (Suga et al., 1993). However, CNP remains low on the plasma concentration in healthy subjects (Stingo et al., 1992). CNP is an endothelium-derived vasodilator agent (Suga et al., 1992), it induces vasodilation in an endothelium-dependent manner (Amin et al., 1996; Andrade et al., 2014) and it can hyperpolarize plasma membrane of vascular smooth muscle (VSM) cell through natriuretic peptide receptor (NPR)-C activation (Chauhan et al., 2003; Garcha and Hughes, 2006). Natriuretic peptides, i.e., atrial natriuretic peptide (ANP), brain natriuretic peptide (BNP), and CNP are able to induce intravascular shedding of glycocalyx and increase vascular permeability (Jacob et al., 2013). Furthermore, the authors suggest that this condition might be present in the pathophysiology of sepsis.

ANP, BNP (Piechota et al., 2009), and CNP (Hama et al., 1994) are elevated on plasma samples from septic subjects. Until now, three different NPR were characterized: NPR-A, NPR-B, and NPR-C (Kone, 2001). ANP and BNP present affinity for NPR-A, whereas CNP is the endogenous agonist for NPR-B. Both NPR-A and NPR-B are associated with particulate guanylyl cylclase (pGC), leading to the intracellular cyclic guanosine-monophosphate (cGMP) formation (Rautureau et al., 2010). ANP, BNP, and CNP have similar affinity for NPR-C, which is associated with G_i_ protein, adenylyl cyclase inhibition, and phospholipase C-β (PLC-β) activation (Matsukawa et al., 1999), as well as activation of NOS induced by calcium increase rather than NOS phosphorylation (Murthy et al., 1998; Costa et al., 2007; Chen et al., 2008; Andrade et al., 2014).

In VSM cells stimulated with LPS or tumor necrosis factor-α (TNF-α), natriuretic peptides increase nitrite formation while the NOS inhibitor reduces CNP responses, suggesting the involvement of NO on CNP effects (Marumo et al., 1995). NPR-A/B antagonist infusion attenuated the hypotension in a sepsis model (Hinder et al., 1997). According to Panayiotou et al. (2010), knockout mice to NPR-A exposed to LPS have low systemic hypotension, and lower levels of NO metabolites, cGMP levels and NOS2 expression compared to wild-type septic animals. Therefore, the contribution of natriuretic peptide system to the vascular dysfunction seems to be evident on sepsis. However, little is known about the role of CNP in this dysfunction and if it could act as a modulator of α_1_-adrenergic vascular contraction on animals septic vessels. The hypothesis of the present work was that CNP induces anti-contractile effect on phenylephrine (PE) contraction in septic vessels, which effect would be dependent of NO and reactive oxygen species (ROS). This study aimed to evaluate the contribution of intracellular NO and ROS, and the role of activation of NPR-B in the CNP-anti-contractile effect on PE-contraction in aortas isolated from rats submitted to CLP surgery to induce sepsis.

## Methods

### Animals

All the procedures were performed in accordance with the standards and policies of the Ethics Committee on Animal Care and Use of the University of São Paulo (#144/2011). Male rats (200 g) were anesthetized with Tribromoethanol (0.25 g.Kg^−1^, i.p.) before cathetherization surgery on femoral artery. On the next day, the MAP was measured for basal values (before surgery), using pressure transducer. Then, the rats were anesthetized with Tribromoethanol (0.25 g.Kg^−1^, i.p.) and randomly submitted to CLP surgery to induce sepsis, with 12 punctures using a 16 gauge-needle on anti-mesenteric border of the cecum (Wichterman et al., 1980; Araújo et al., 2011), or the control group was submitted to a medial laparotomy only (Sham). Survival and MAP were evaluated for 24 h after surgeries. Animals were maintained on standard rat chow and water.

### Functional studies by vascular reactivity

Sham and CLP rats were killed by decapitation under anesthesia (inhaled Isoflurane) and thoracic aorta and resistance mesenteric artery (RMA) were used to measure the isometric tension.

#### Vascular reactivity studies on aorta

Aorta was cut into rings (4 mm) and placed between two stainless-steel stirrups and connected to an isometric force transducer to measure tension. Cumulative concentration-effect curves were constructed to PE (0.1 nmol.L^−1^−10 μmol.L^−1^) in intact endothelium rat aortas. These curves were obtained in the absence (CO) or after 30 min incubation with CNP (10 nmol.L^−1^), N^ω^-nitro-L-arginine methyl ester hydrochloride (L-NAME, 100 μmol.L^−1^), N^ω^-propyl-L-arginine (50 nmol.L^−1^), 1400 W (10 nmol.L^−1^), Tiron (100 μmol.L^−1^), polyethylene glycol-Catalase (PEG-Catalase, 250 U.mL^−1^), Anantin (Ana, 0.1 μmol.L^−1^ or 1 μmol.L^−1^) alone or in the combination of each inhibitor/antagonist and CNP. The PE potency (*p*D_2_) and contractile maximum effect (ME) were evaluated.

#### Vascular reactivity on RMA

RMA were isolated and cut into rings (2 mm) using a dissection magnifier in cold Krebs solution for small arteries. Only the second branch of RMA with 200–350 μm of internal circumference were used. A tungsten wire (40 μm) was passed into the vessel and tied in myograph to resistance vessels and another was connected to isometric force transducer. Cumulative concentration-effect curves were constructed to PE (0.1 nmol.L^−1^−100 μmol.L^−1^) in intact endothelium RMA in the absence or in the presence of CNP (10 nmol.L^−1^, 30 min). The area under the curve (AUC) of PE contraction was evaluated.

### Measurement of plasma NO metabolites

Cardiac blood samples were collected from Sham or CLP rats in heparin-containing tubes. Plasma aliquots of Sham or CLP rats were analyzed for their nitrite content using an ozone-based reductive chemiluminescence assay (Pinheiro et al., 2012). The plasma NOx concentration was determined by using the Griess reaction (Pinheiro et al., 2012).

### Western blotting analysis

Protein expression of NOS2, NOS3, NPR-B, or Nox1 were analyzed in Sham or CLP rat aorta homogenate. Membranes were incubated with mouse primary antibody anti-NOS2 (1:2500), or anti-NOS3 (1:2500), and rabbit primary antibody anti-NPR-B (1:5000), or anti-Nox1 (1:2000), overnight at 4°C. Then, membranes were incubated with a HRP-conjugated goat anti-rabbit (1:5000) or goat anti-mouse secondary antibody (1:5000) for 60 min at room temperature. Protein bands were visualized by means of chemiluminescence. Protein expression levels were normalized by mouse anti-β-actin (1:2000).

### Confocal microscopy analysis

Aorta was isolated from Sham or CLP rats and frozen on cryoprotection liquid. Sheets were prepared with 10 μm of thickness and immunofluorescence was performed to evaluate endogenous CNP (1:100) or NPR-C (1:100) expression. The secondary antibody mouse anti-goat Alexafluor 647 (1:1000) or sheep anti-rabbit Alexafluor 647 (1:1000) was incubated for 1 h, at room temperature. Fluoroshield® with DAPI was applied overnight at 4°C. Images were acquired using confocal microscopy.

### Lucigenin chemiluminescence analysis

Vascular nicotinamide adenine dinucleotide phosphate (NADPH)-dependent ROS production was assessed in intact-endothelium aorta from Sham or CLP rats, previously stimulated with PE (0.1 μmol.L^−1^) in the absence or in the presence of CNP (10 nmol.L^−1^, 30 min) or not stimulated (basal). After that, aortas were collected and frozen on liquid nitrogen. The luminescence was measured using Single Tube Luminometer Berthold FB12, at 37°C. Data were presented as relative luminescence units (RLU).mg^−1^.min^−1^.

### Quantitative polymerase chain reaction (qPCR) analysis

Total ribonucleic acid (RNA) was extracted from aorta homogenates isolated from Sham or CLP rats with TRIzol® reagent according to manufacturer's instruction. The cDNA was synthesized with 1 μg of RNA using anchored random hexadeoxynucleotide in the reaction conditions for transcriptor First-strand cDNA Synthesis Kit. qPCR was performed using SYBR® FAST qPCR kit Master Mix Universal with 50 nmol.L^−1^ of primers for PKCα (protein kinase-Cα) or GAPDH (glyceraldehyde-3-phosphate dehydrogenase). The samples were incubated for 2 min at 50°C and 10 min at 95°C followed by 40 cycles at 95°C for 15 s and 60°C for 1 min.

### Drugs and solutions

Drugs and solutions are presented on Supplemental Material.

### Statistical analysis

Results are presented as mean ± S.E.M. Each experimental *n* represents samples isolated from different animals. Comparisons between groups were conducted by the Student's *t*-test, One-way analysis of variance followed by Newman-Keuls, or Two-way analysis of variance using Bonferroni correction for multiple comparisons, as appropriate. The level of statistical significance was defined as *P* < 0.05.

## Results

### Survival and mean arterial pressure analysis

**Figure 1:**
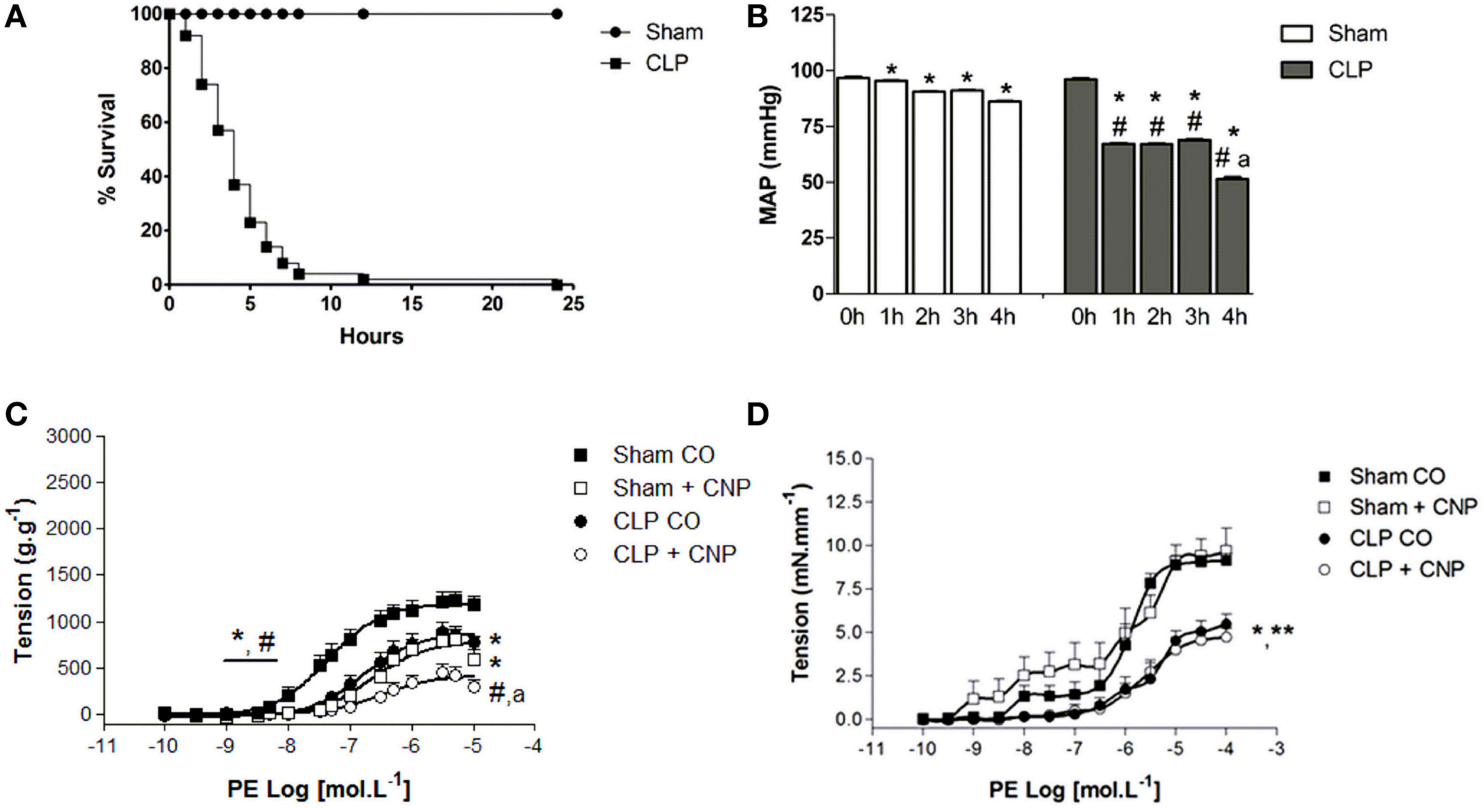
**Percentage of survival analysis (A) of control-operated (Sham *n* = 20) and CLP (*n* = 30) rats**. Mean arterial pressure **(B)** (MAP, mmHg) before (0 h) or after (from 1 to 4 h) surgeries in Sham (*n* = 6) or CLP (*n* = 6) rats. Cumulative concentration-effect curves to phenylephrine (PE) in the absence or in the presence of CNP (10 nmol.L^−1^; Sham *n* = 8; CLP *n* = 8) in aorta **(C)** or resistance mesenteric artery (RMA, Sham *n* = 6; CLP *n* = 7) **(D)** with endothelium, from Sham or CLP rats. Data are represented as mean ± S.E.M. In **(B)**: ^*^different from 0 h; ^#^different from Sham respective time; ^a^different from CLP 1, 2, or 3 h. In **(C)**: ^*^different from Sham CO; ^#^different from CLP CO; ^a^different from Sham CNP. In **(D)**: ^*^different from Sham CO; ^**^different from Sham CNP. Each experimental *n* represents samples isolated from different animals. Two-way Anova, Bonferroni correction to evaluate interaction factor; One-way Anova, *pos-hoc* Newman-Keuls to three or more comparisons (*P* < 0.05).

Sham rats survival rate was 100% at 24 h after surgery (*n* = 20). CLP rats had lower survival rate than Sham, and 4 h after the CLP surgery it was 37% of survival, reaching no longer than 2% of survival at 24 h (*n* = 30) (Figure 1). Basal MAP was similar in both groups (Sham: 96.7 ± 0.6 mmHg, *n* = 6; CLP: 96.1 ± 0.4 mmHg, *n* = 6). Four hours after the surgeries, MAP decreased in both groups which values were lower on CLP (51.4 ±0.9 mmHg, *n* = 6) than in Sham rats (86.2 ± 0.3 mmHg, *n* = 6) (Figure 1).

### PE induced contraction and CNP induced anti-contractile effect in aorta but not in RMA

The ME and potency (*p*D_2_) to PE were lower on CLP (ME: 753 ± 47 g.g^−1^, *p*D_2_: 6.74 ± 0.14, *n* = 11) than in Sham aortas (ME: 1232 ± 30 g.g^−1^, *p*D_2_: 7.32 ± 0.10, *n* = 11) (Figure 1). Similarly, in RMA, PE-induced contraction was lower in CLP (4.73 ± 0.52 mN.mm^−1^; *n* = 7) than in Sham (9.68 ± 1.30 mN.mm^−1^; *n* = 6). The AUC of PE-induced contraction on RMA was also lower in CLP (9.55 ± 0.86) than in Sham (24.27 ± 2.75). CNP induced anti-contractile effect in aortas but not in RMA (Figure 1). CNP reduced the PE-induced contraction in Sham aortas (ME: 877 ± 25 g.g^−1^; *p*D_2_: 6.68 ± 0.10; *n* = 8). However, only ME was decreased by CNP in CLP aortas (ME: 365 ± 74 g.g^−1^). CNP anti-contractile effect was higher in CLP than Sham aortas.

### NPR-C was decreased in CLP compared to Sham aortas

**Figure 2:**
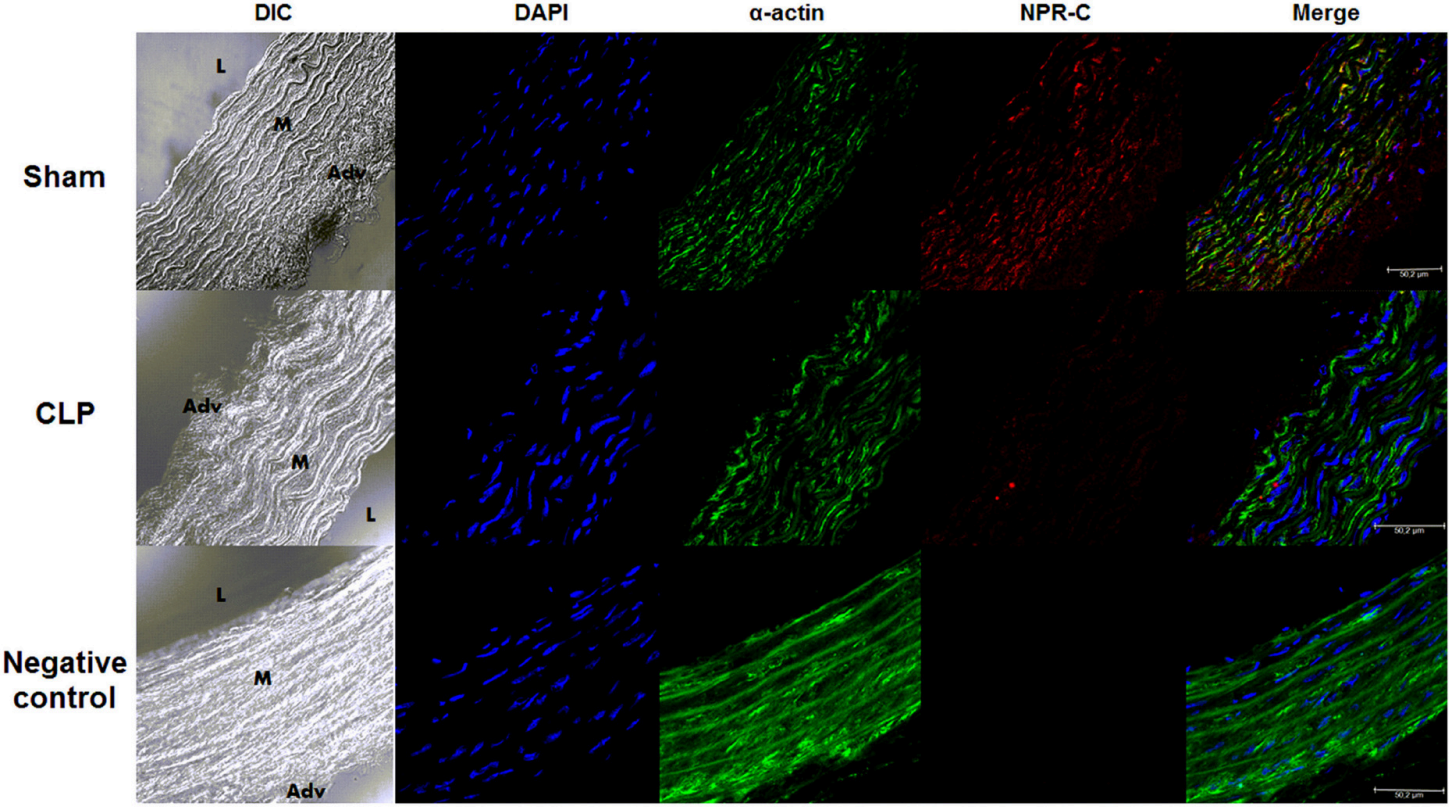
**NPR-C staining in vascular layers in Sham or CLP rat aortas**. Photomicrograph of immunofluorescence of NPR-C staining in Sham (*n* = 6) or CLP (*n* = 6) rat aortas. DIC represents dichroic contrast phase, DAPI represents nuclei staining, α-actin from α-actin smooth muscle-FITC staining. Bar represents 50 μm. *L, M*, and *Adv* represent lumen, media layer, and adventitia, respectively. Each experimental *n* represents samples isolated from different animals.

As shown in the photomicrographs (Figure 2) and in the measurement of fluorescence intensity (FI) emitted by anti-NPR-C antibody, the NPR-C expression was lower in CLP (4.35 ± 0.46 U, *n* = 6) compared to Sham aortas (7.81 ± 0.80 U, *n* = 6).

### Plasma NO and protein expression of NOS3 and NOS2 were increased, and NPR-B was not changed in CLP aortas

**Figure 3:**
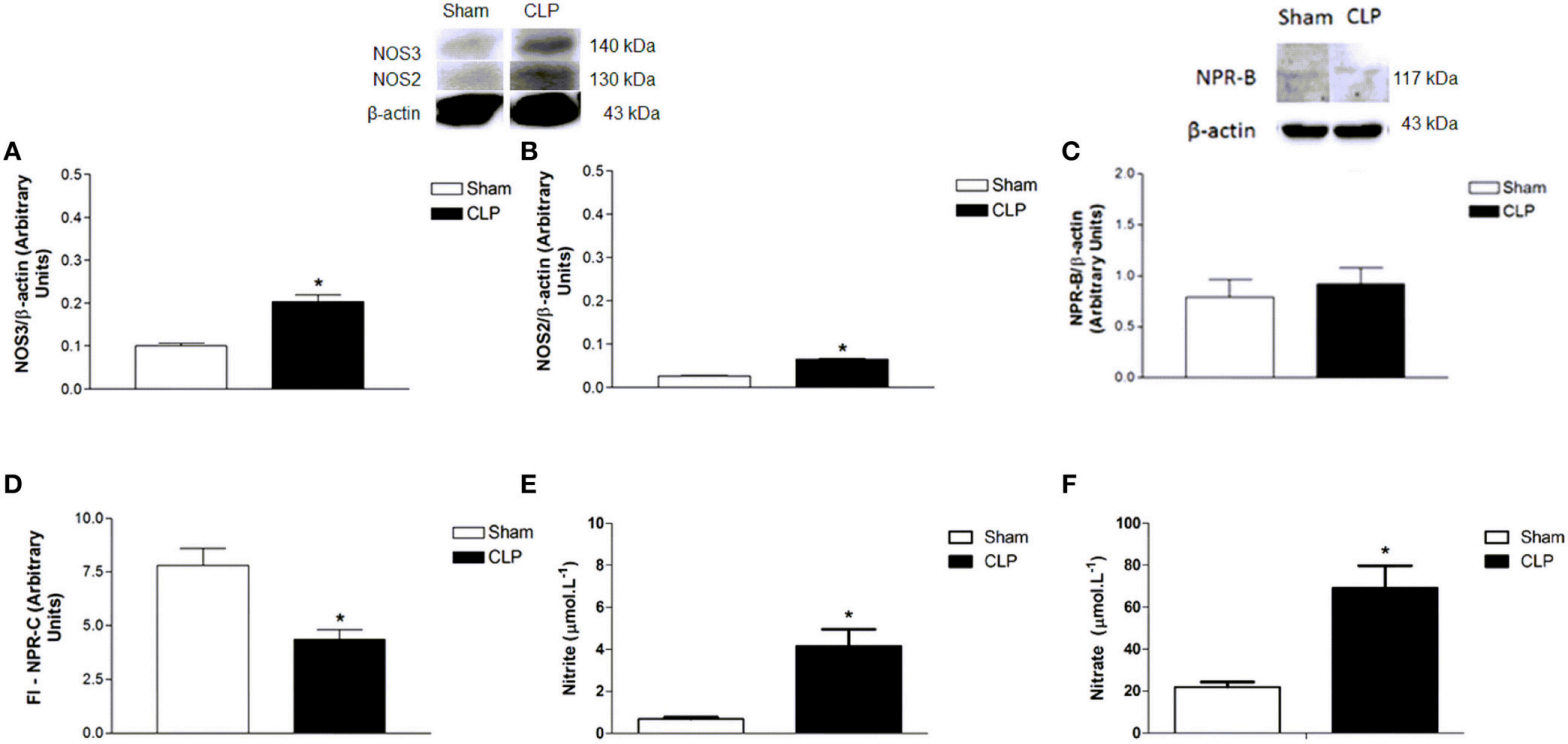
**Protein expression of NOS3 (A, Sham *n* = 4; CLP *n* = 4), NOS2 (B, Sham *n* = 4; CLP *n* = 4) and NPR-B (C, Sham *n* = 4; CLP *n* = 6) on aorta homogenate of Sham or CLP rats**. Measurement **(D)** of fluorescence intensity (FI) emitted by anti-NPR-C antibody in Sham (*n* = 6) or CLP (*n* = 6) rat aortas. Measurement of plasma nitrite (**E**, Sham *n* = 4; CLP *n* = 4) and nitrate (**F**, Sham *n* = 7; CLP *n* = 7). Western blotting data were normalized by β-actin, and presented as mean ± S.E.M. ^*^different from Sham. Each experimental *n* represents samples isolated from different animals. Two-way Anova, Bonferroni correction to evaluate interaction factor; One-way Anova, *pos-hoc* Newman-Keuls to three or more comparisons; Student *t*-test to compare two different groups (*P* < 0.05).

NOS3 protein expression in CLP aorta was greater (0.20 ± 0.02 U, *n* = 4) than in Sham aorta (0.10 ± 0.01, *n* = 4) (Figure 3). Similarly, NOS2 protein expression in CLP aorta was higher (0.06 ± 0.002 U, *n* = 4) than in Sham aorta (0.03 ± 0.001 U, *n* = 4). However, NPR-B protein expression was not different between Sham (0.79 ± 0.07 U, *n* = 4) and CLP (0.92 ± 0.06 U, *n* = 6). Plasma NO metabolites such as nitrite and nitrate were higher on CLP (nitrite: 4.16 ± 0.79 μmol.L^−1^, *n* = 4; nitrate: 69.12 ± 10.58 μmol.L^−1^, *n* = 7) than in Sham (nitrite: 0.69 ± 0.09 μmol.L^−1^, *n* = 4; nitrate: 21.97 ± 2.38 μmol.L^−1^, *n* = 7) rats.

### NPR-C was lower and NPR-B was not changed in CLP in relation to Sham aortas

NPR-B protein expression was not different between Sham (0.79 ± 0.07 U, *n* = 4) and CLP (0.92 ± 0.06 U, *n* = 6), whereas NPR-C expression was lower in CLP (4.35 ± 0.46 U, *n* = 6) aortas as compared to Sham (7.81 ± 0.80 U, *n* = 6), as shown in Figure 3.

### CNP-induced anti-contractile effect was dependent on NOS activity in rat aortas

**Figure 4:**
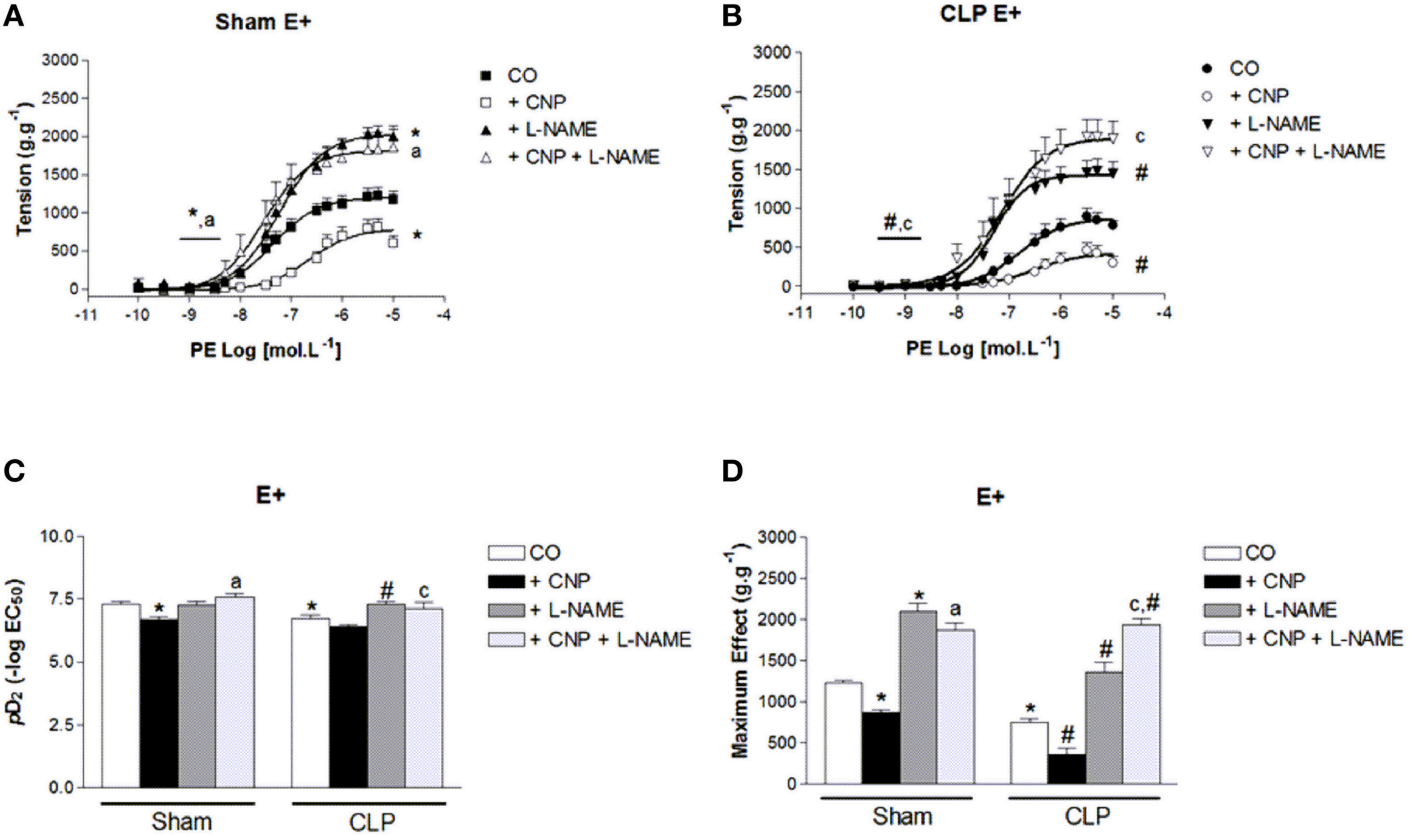
**Cumulative concentration-effect curves to phenylephrine (PE) in the absence (CO) or presence of CNP (10 nmol.L^−1^); non-selective NOS-inhibitor L-NAME (100 μmol.L^−1^) or CNP; and L-NAME in Sham (A) (*n* = 11) or CLP rat aorta (B) (*n* = 12)**. *p*D_2_ values **(C)** of PE curves of Sham or CLP rat aorta. Maximum effect values **(D)** (g.g^−1^) of PE curves of Sham or CLP rat aorta. Data are presented as mean ± S.E.M. ^*^different from Sham CO; ^#^different from CLP CO; ^a^different from Sham CNP; ^c^different from CLP CNP. Each experimental *n* represents samples isolated from different animals. Two-way Anova, Bonferroni correction to evaluate interaction factor; One-way Anova, *pos-hoc* Newman-Keuls to three or more comparisons (*P* < 0.05).

PE-induced contraction was increased by the non-selective inhibition of NOS with L-NAME in Sham (2097 ± 97 g.g^−1^, *n* = 11) and CLP (1365 ± 116 g.g^−1^, *n* = 12) aortas. But it was still lower in CLP aortas (Figure 4). However, *p*D_2_ value increased in CLP aortas (7.30 ± 0.09). CNP-anti-contractile effect on PE-induced contraction was reversed by L-NAME in both groups (Sham: 1876 ± 83 g.g^−1^, *n* = 11; CLP: 1936 ± 82 g.g^−1^, *n* = 12). L-NAME and CNP increased *p*D_2_ values in Sham (7.58 ± 0.16) and in CLP (7.10 ± 0.28) aortas.

### NOS1 and NOS2 also contributes to CNP-induced anti-contractile effect in rat aortas

**Figure 5:**
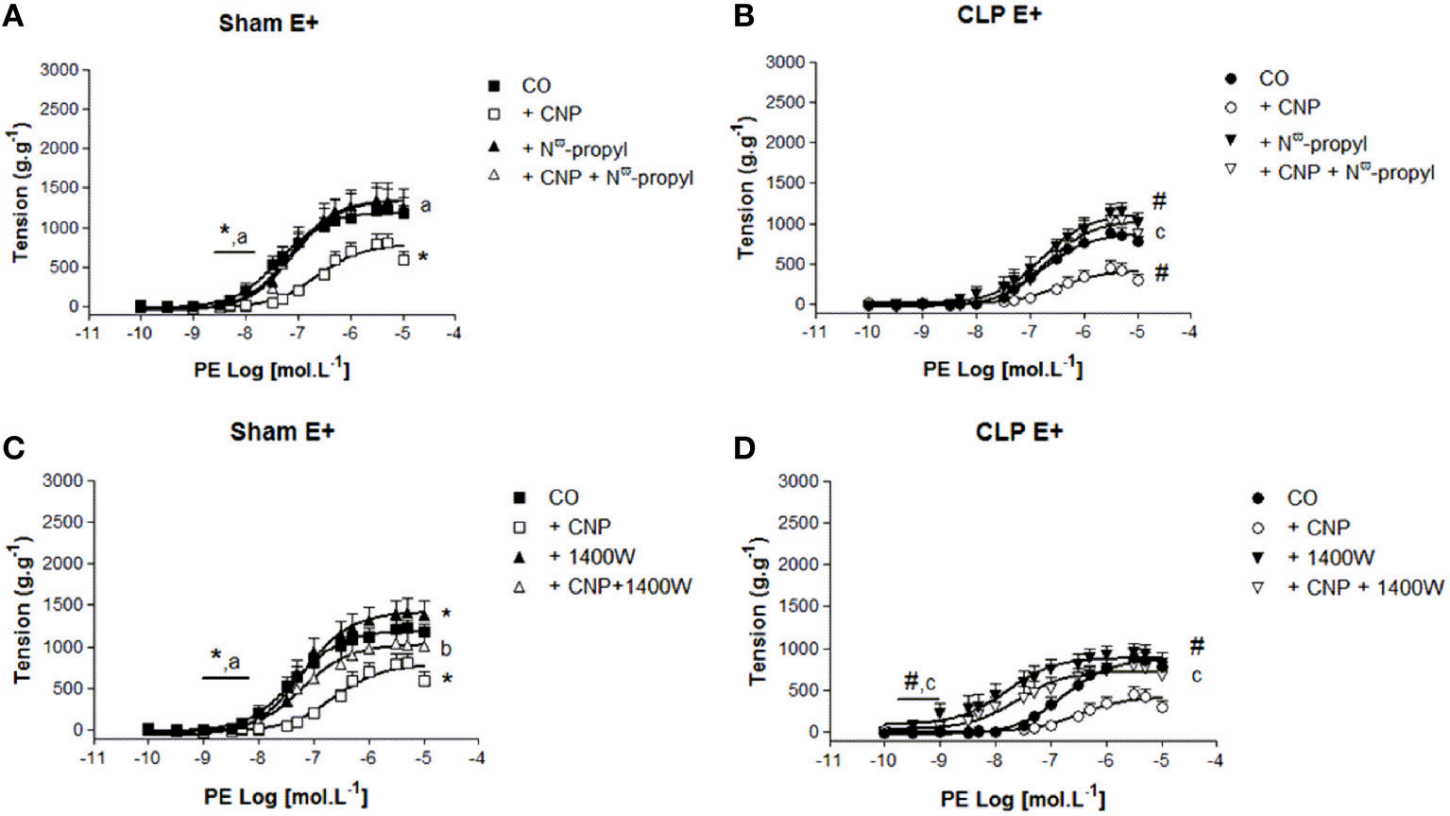
**Cumulative concentration-effect curves to phenylephrine (PE) in the absence (CO) or presence of CNP (10 nmol.L^−1^); selective NOS1-inhibitor N^ω^-propyl-L-arginine (N^ω^-propyl, 50 nmol.L^−1^) or CNP + N^ω^-propyl in Sham (A) (*n* = 9) or CLP rat aorta (B) (*n* = 5)**. Cumulative concentration-effect curves to PE in the absence (CO) or presence of CNP (10 nmol.L^−1^); selective NOS2-inhibitor 1400 W (100 nmol.L^−1^) or CNP + 1400 W in Sham **(C)** (*n* = 8) or CLP rat aorta **(D)** (*n* = 6). Data are presented as mean ± S.E.M. ^*^different from Sham CO; ^#^different from CLP CO; ^a^different from Sham CNP; ^b^different from Sham + 1400 W; ^c^different from CLP CNP. Each experimental *n* represents samples isolated from different animals. Two-way Anova, Bonferroni correction to evaluate interaction factor; One-way Anova, *pos-hoc* Newman-Keuls to three or more comparisons (*P* < 0.05).

The selective NOS1 inhibitor N^ω^-propyl did not modify the potency to PE in Sham (7.20 ± 0.10, *n* = 8) or CLP (7.05 ± 0.20, *n* = 7) aortas (Figure 5). This inhibitor increased ME to PE only in CLP aortas (1109 ± 79 g.g^−1^), whereas this contraction remained unchanged in Sham (1349 ± 53 g.g^−1^). CNP-anti-contractile effect on PE-induced contraction was reversed by N^ω^-propyl in Sham (1342 ± 67 g.g^−1^, *n* = 9) or CLP (1060 ± 126 g.g^−1^, *n* = 5) aortas (Figure 5). PE-induced maximum contraction in CLP (1060 ± 126 g.g^−1^, *n* = 5) was lower than in Sham (1342 ± 67 g.g^−1^) aortas in the presence of CNP and N^ω^-propyl. In addition, CNP-anti-contractile effect on PE potency was reversed in Sham (7.08 ± 0.09) and it was not modified in CLP (6.71 ± 0.21) aortas.

The selective NOS2 inhibitor 1400 W, increased PE *p*D_2_ value in CLP (8.04 ± 0.34) and the ME to PE in Sham (1427 ± 65 g.g^−1^, *n* = 7) and CLP (954 ± 52 g.g^−1^, *n* = 7), which effect was lower in CLP than in Sham aortas (Figure 5). The double exposure CNP and 1400 W increased PE potency in Sham (7.19 ± 0.15, *n* = 8) and CLP aortas (7.63 ± 0.34, *n* = 6). The ME to PE was greater in CLP (789 ± 86 g.g^−1^) after the double exposure to CNP and 1400 W, but not in Sham aortas (941 ± 57 g.g^−1^).

### CNP-induced anti-contractile effect was dependent on ROS in rat aortas

**Figure 6:**
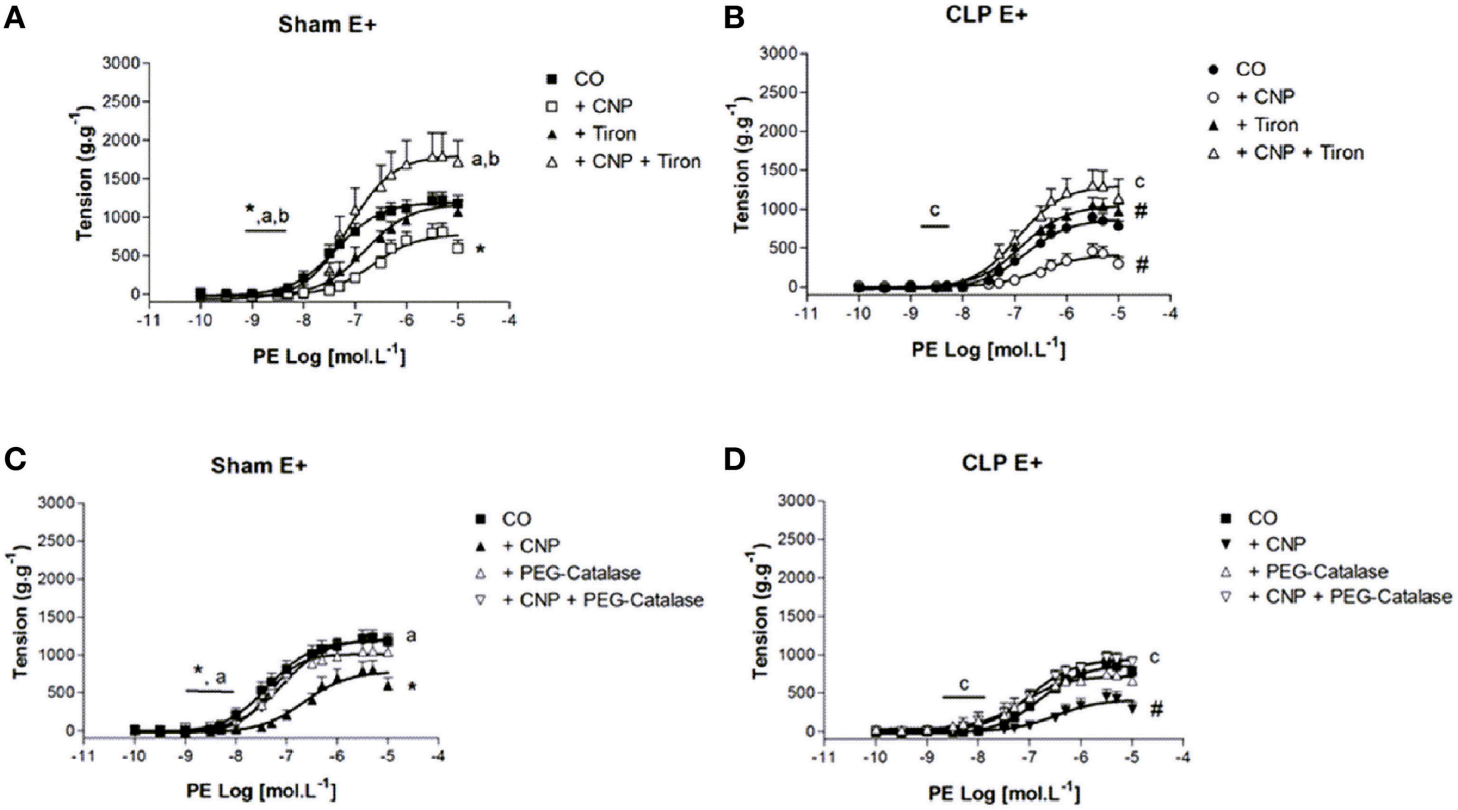
**Cumulative concentration-effect curves to phenylephrine (PE) in the absence (CO) or presence of CNP (10 nmol.L^−1^); ${\text{O}}_{2}^{–}$ scavenger Tiron (100 μmol.L^−1^) or CNP + Tiron in Sham (A) (*n* = 6) or CLP rat aorta (B) (*n* = 13)**. Cumulative concentration-effect curves to PE in the absence (CO) or presence of CNP (10 nmol.L^−1^); intracellular H_2_O_2_ breakdown agent PEG-catalase (250 U.mL^−1^) or CNP + PEG-catalase in Sham **(C)** (*n* = 5) or CLP rat aorta **(D)** (*n* = 11). Data are presented as mean ± S.E.M. ^*^different from Sham CO; ^#^different from CLP CO; ^a^different from Sham CNP; ^b^different from Sham Tiron; ^c^different from CLP CNP. Each experimental *n* represents samples isolated from different animals. Two-way Anova, Bonferroni correction to evaluate interaction factor; One-way Anova, *pos-hoc* Newman-Keuls to three or more comparisons (*P* < 0.05).

The superoxide (${\text{O}}_{2}^{–}$) scavenger Tiron reduced the potency to PE in Sham (6.70 ± 0.15) but not in CLP (6.69 ± 0.20) aortas (Figure 6). However, ME to PE was enhanced on CLP (1034 ± 70 g.g^−1^) aortas. Moreover, CNP-anti-contractile effect on PE contraction was reversed by Tiron on *p*D_2_ values (Sham: 7.18 ± 0.10, *n* = 6; CLP: 7.01 ± 0.13, *n* = 13) and ME (Sham: 1822 ± 101 g.g^−1^, *n* = 6; CLP: 1328 ± 104 g.g^−1^, *n* = 13). Although ME to PE in CLP aortas in the presence of CNP and Tiron induced a greater value compared to CNP alone, this value remained lower than that one on Sham aortas.

The intracellular breakdown of hydrogen peroxide (H_2_O_2_) with PEG-Catalase did not change the contractile response to PE in both groups (Figure 6). However, the presence of PEG-Catalase and CNP reversed CNP-anti-contractile effect on PE potency (Sham: 7.07 ± 0.10, *n* = 5; CLP: 7.20 ± 0.25, *n* = 11) and ME (Sham: 1199 ± 60 g.g^−1^, *n* = 5; CLP: 970 ± 80 g.g^−1^, *n* = 11).

### CNP-induced anti-contractile effect was dependent on NPR-B activation and PE contraction was modulated by NPR-B in rat aortas

**Figure 7:**
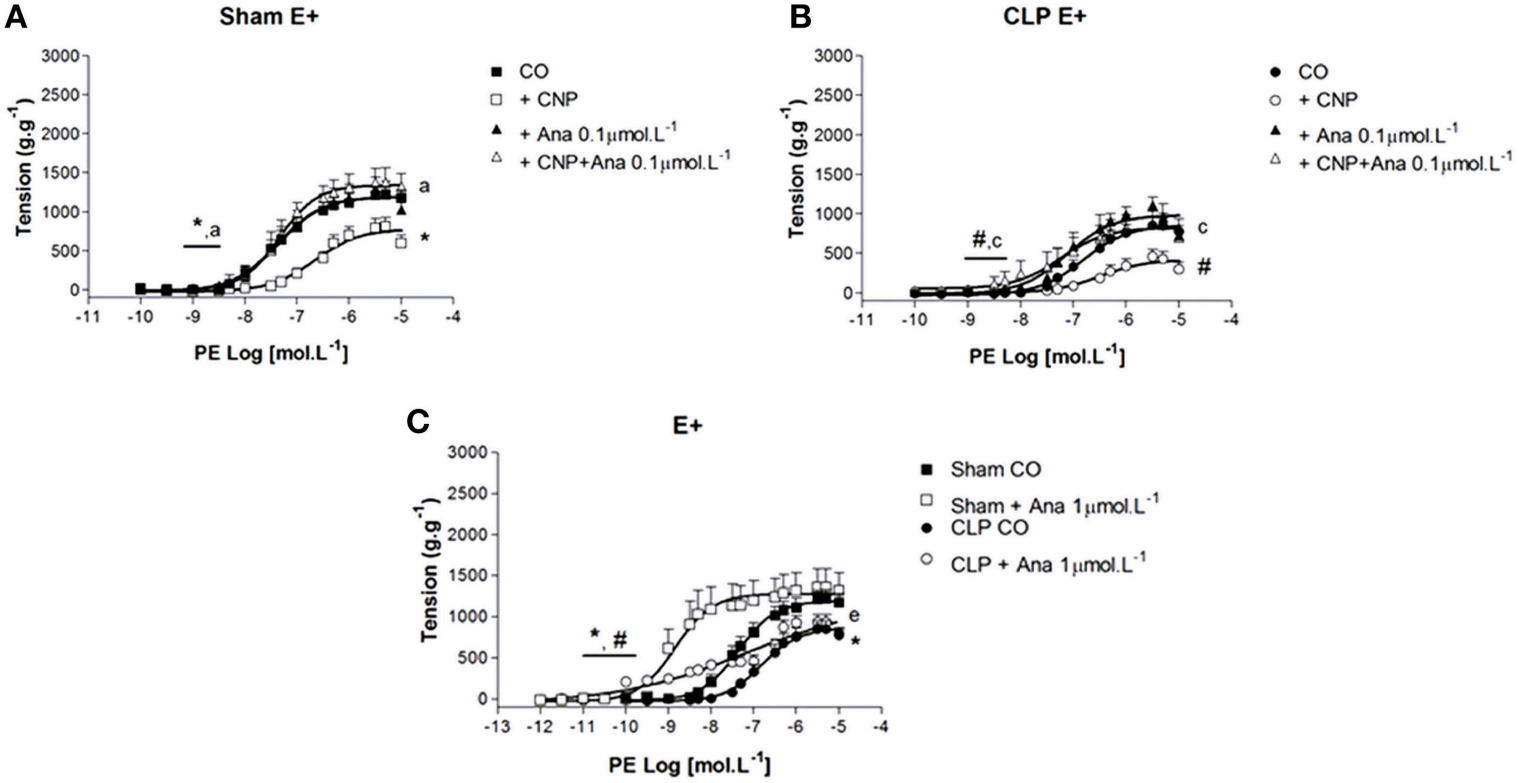
**Cumulative concentration-effect curves to phenylephrine (PE) in the absence (CO) or presence of CNP (10 nmol.L^−1^); NPR-A/B antagonist Anantin (Ana) in lower concentration (0.1 μmol.L^−1^) or CNP + Ana in Sham (A) (*n* = 8) or CLP rat aorta (B) (*n* = 13)**. Cumulative concentration-effect curves to PE in absence or in the presence of Ana in higher concentration (1 μmol.L^−1^) in Sham (*n* = 5) or CLP rat aorta (*n* = 11) **(C)**. Data are presented as mean ± S.E.M. ^*^different from Sham CO; ^#^different from CLP CO; ^a^different from Sham CNP; ^c^different from CLP CNP; ^e^different from Sham Ana. Each experimental *n* represents samples isolated from different animals. Two-way Anova, Bonferroni correction to evaluate interaction factor; One-way Anova, *pos-hoc* Newman-Keuls to three or more comparisons (*P* < 0.05).

The non-selective NPR-A/B antagonist Anantin (0.1 μmol.L^−1^) did not modify the PE *p*D_2_ values (Sham: 7.23 ± 0.23, *n* = 8; CLP: 7.05 ± 0.19, *n* = 13). However, it increased ME to PE in CLP (1093 ± 107 g.g^−1^) but not in Sham (1198 ± 114 g.g^−1^) aorta (Figure 7). CNP-anti-contractile effect was reversed in Sham and CLP (*p*D_2_: Sham 7.50 ± 0.10; CLP: 7.35 ± 0.30; ME: Sham 1345 ± 115 g.g^−1^, *n* = 8; CLP: 891 ± 86 g.g^−1^, *n* = 13) aortas. When Anantin was used at the concentration of 1 μmol.L^−1^, PE-induced contraction was potentiated in both groups (Sham: 8.49 ± 0.30, *n* = 5; CLP: 8.06 ± 0.80, *n* = 11), but it did not change ME to PE. In addition, the PE *p*D_2_ values in the presence of 1 μmol.L^−1^Anantin were greater than the values observed with 0.1 μmol.L^−1^Anantin in Sham.

### Endogenous expression of CNP was lower in endothelium and higher in VSM layer in CLP than in Sham rat aortas

**Figure 8:**
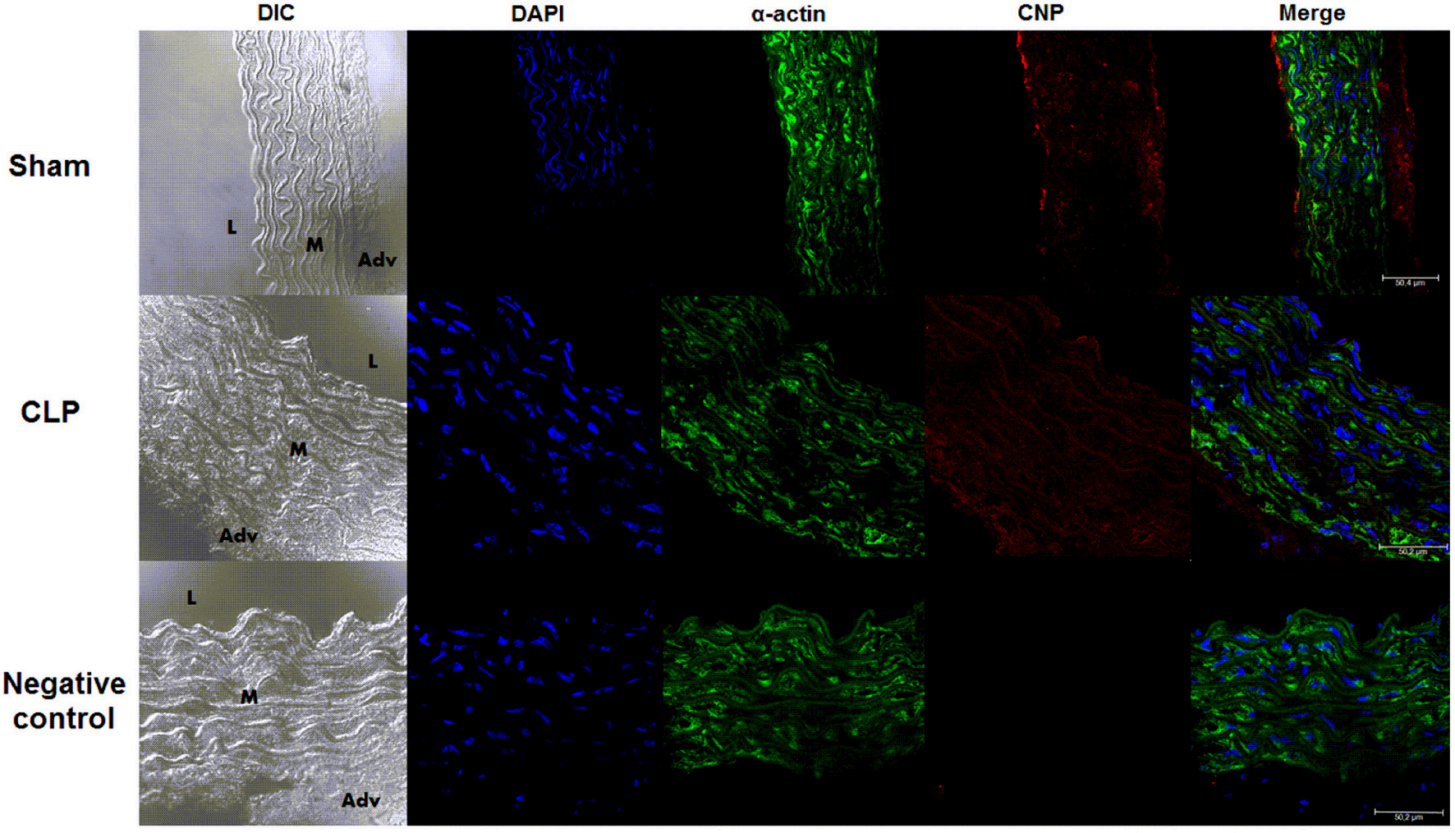
**Endogenous CNP staining in vascular layers**. Photomicrograph of immunofluorescence of CNP staining in Sham (*n* = 4) or CLP (*n* = 6) rat aortas. DIC represents dichroic contrast phase, DAPI represents nuclei staining, α-actin from α-actin smooth muscle-FITC staining. Bar represents 50 μm. *L, M*, and *Adv* represent lumen, media layer, and adventitia, respectively. Each experimental *n* represents samples isolated from different animals.

**Figure 9:**
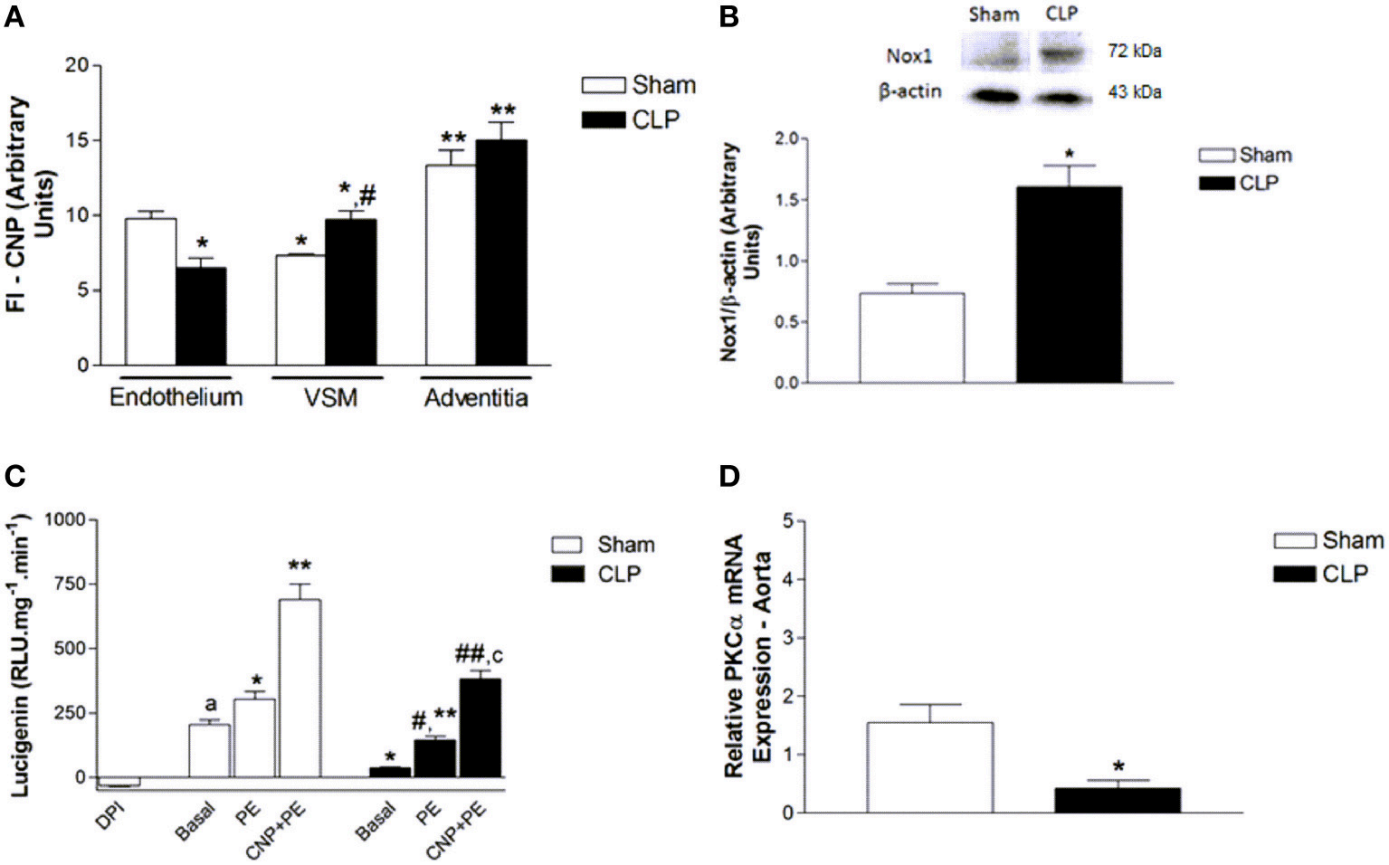
**Measurement of FI emitted by anti-CNP antibody (A) in Sham (*n* = 4) or CLP (*n* = 6) rat aortas**. Protein expression of Nox1 **(B)** and NADPH-dependent reactive oxygen species (ROS) production **(C)** in aorta homogenate from Sham (*n* = 4) or CLP (*n* = 4) rats. ROS production (relative luminescence units.mg^−1^.min^−1^) was evaluated in the absence (basal, Sham *n* = 5; CLP *n* = 3) or in the presence of PE (10 μmol.L^−1^, Sham *n* = 8; CLP *n* = 5) or CNP (10 nmol.L^−1^) + PE (10 μmol.L^−1^, Sham *n* = 6; CLP *n* = 5). Negative control was evaluated in the presence of non-selective inhibitor of flavoproteins DPI (10 μmol.L^−1^, *n* = 3) in Sham aorta. Relative PKCα mRNA expression **(D)** in aorta homogenate from Sham (*n* = 4) or CLP (*n* = 4) rats. Western blotting data were normalized by β-actin. Quantitative PCR analysis was normalized by GAPDH (glyceraldehyde-3-phosphate dehydrogenase) mRNA expression. Data are presented as mean ± S.E.M. In **(B)**: ^*^different from Sham endothelium; ^#^different from CLP endothelium; ^**^different from VSM from respective groups. In **(C)**: ^*^different from Sham. In **(D)**: ^*^different from Sham basal; ^#^different from CLP basal; ^a^different from Sham DPI; ^**^different from Sham PE; ^*##*^different from CLP PE; ^c^different from Sham PE CNP. In **(E)**: ^*^different from Sham. Each experimental *n* represents samples isolated from different animals. Two-way Anova, Bonferroni correction to evaluate interaction factor; One-way Anova, *pos-hoc* Newman-Keuls to three or more comparisons; Student *t*-test to compare two different groups (*P* < 0.05).

The endothelial expression of endogenous CNP, as shown in the photomicrographs (Figure 8) and the measurement of FI of anti-CNP antibody (Figure 9), was lower in CLP (6.50 ± 0.66 U, *n* = 6) than in Sham aortas (9.78 ± 0.50 U, *n* = 4). However, CNP expression on VSM was higher in CLP (9.72 ± 0.60 U, *n* = 6) compared to Sham aortas (7.32 ± 0.11 U, *n* = 4). It was not different in the adventitia isolated from Sham (13.35 ± 1.00 U, *n* = 4) and CLP aortas (15.02 ± 1.20 U, *n* = 6). It can be noted that endogenous CNP is higher in the adventitia than in the endothelium which is higher than in the VSM in Sham. CNP is higher in adventitia than in the VSM that is higher than in the endothelium in CLP aortas.

### PE and CNP enhanced NADPH-dependent ROS production

Nox1 expression was higher in CLP (1.61 ± 0.17 U, *n* = 4) than in Sham aortas (0.73 ± 0.08 U, *n* = 4). Basal NADPH-dependent ROS production was lower in CLP (36.95 ± 3.27 RLU.mg^−1^.min^−1^, *n* = 3) than in Sham aortas (204.07 ± 20.61 RLU.mg^−1^.min^−1^, *n* = 5). PE increased NADPH-dependent ROS production in Sham (303.69 ± 30.78 RLU.mg^−1^.min^−1^, *n* = 8) and CLP aortas (145.66 ± 14.05 RLU.mg^−1^.min^−1^, *n* = 5). CNP and PE further increased NADPH-dependent ROS production in Sham (690.34 ± 60.05 RLU.mg^−1^.min^−1^, *n* = 6) and CLP rat aortas (382.78 ± 32.45 RLU.mg^−1^.min^−1^, *n* = 5). However, all the values obtained in CLP were lower than in Sham. The non-selective flavoproteins inhibitor DPI, which was used as a negative control, inhibited the basal NADPH-dependent ROS production in Sham rat aorta (−32.44 ± 2.91 RLU.mg^−1^.min^−1^, *n* = 3) (Figure 9).

### PKCα mRNA expression was lower in CLP than in Sham rat aortas

The relative PKCα mRNA expression (Figure 9) was lower in CLP (0.43 ± 0.13, *n* = 4) than in Sham (1.55 ± 0.31, *n* = 4) aortas.

## Discussion

The main findings of this work were: (i) CNP induced anti-contractile effect on PE contraction in rat aortas but not in rat RMA, (ii) this CNP anti-contractile effect was dependent on NOS activity, ROS production and NPR-B activation in Sham and CLP aortas, (iii) endogenous CNP was reduced on endothelium and increased on the VSM cells in CLP aortas, (iv) NPR-C expression was low on VSM layer of CLP aortas, (v) basal NPR-B activity was able to modulate PE-induced vasoconstriction, and (vi) basal NADPH-dependent ROS production was low in CLP aortas, and PE and CNP increased ROS production. It is the first report that CNP can modulate PE contraction, which effect was higher in CLP than Sham aortas.

CLP experimental sepsis model represents clinical conditions observed on patients with intestinal puncture, developing severe sepsis within few hours or some days (Lundblad et al., 1995). Moreover, it was observed that CLP group had a pronounced systemic hypotension and low survival rate, corroborating with findings from Tyml et al. (1998), Liaw et al. (2005), and Tsao et al. (2010). However, we have shown that Sham rats presented lower hypotensive effect than CLP group. According to Jong et al. (2002) general anesthetics can change cardiovascular parameters. Therefore, in our study, the decreased MAP observed on Sham rats could be due to tribromoethanol effect. Besides systemic hypotension showed on CLP rats, it was noted that PE-induced contraction on aorta and RMA was lower in CLP than in Sham rats.

In accordance to Vromen et al. (1996) and Wang et al. (2004), in CLP rat aortas there is low vasoconstriction induced by norepinephrine. Silva-Santos et al. (2002) noted LPS-exposure of aortas during 12 h, at isolated organ bath, reduced contraction to PE. Usually, such reduced contraction to different agonists have been associated with larger NO production by NOS1 and/or NOS2 (Vromen et al., 1996; Silva-Santos et al., 2002; Wang et al., 2004; Enkhbaatar et al., 2009; Tsao et al., 2010; Nardi et al., 2014), oxidative stress (Tsao et al., 2010), and greater inflammatory mediators production (Panayiotou et al., 2010). Enkhbaatar et al. (2009) showed that specific NOS1 inhibitor, called as ZK234238, attenuated hypotension and the fall in systemic vascular resistance, inhibited the increase in plasma nitrate/nitrite, and attenuated inflammatory markers such as myeloperoxidase activity, IL (interleukin)-6 mRNA, and reactive nitrogen species. Furthermore, Nardi et al. (2014) demonstrated that the treatment with 7-nitroindazole, another NOS1 inhibitor, reverted the hyporesponsivity to PE and norepinephrine, and reduced the production of cGMP stimulated by PE in CLP rat aortas. Our results are in agreement with the others and it was noted raise on plasma NO levels, as nitrite and nitrate, and raise on protein expression of NOS2 and NOS3 in CLP rat aortas. Moreover, the non-selective NOS inhibitor L-NAME, or the selective NOS1 inhibitor N^ω^-propyl, or the selective NOS2 inhibitor 1400 W, increased PE contraction in both groups; but PE-induced maximum contraction was still lower in CLP aortas, suggesting additional mechanisms which are responsible for PE hyporresponsivity.

CNP-induced anti-contractile effect was greater in CLP than in Sham aortas. However, we did not observe CNP-induced modulation of PE contraction in RMA from both groups, using a single concentration of CNP in these studies. Villar et al. (2007) showed CNP-induced relaxation in RMA isolated from rats that was dependent on NPR-C activation. Besides, this group demonstrated all the three NPRs in rat aorta and RMA. We cannot exclude the possibility that higher CNP concentration could induce the anti-contractile effect on these resistance vessels, since the contraction elicited by PE needed higher concentration in RMA than in aorta. Since this CNP anti-contractile effect was observed on aortas at low concentration (10 nmol.L^−1^), we decided to continue to investigate this vessel. CNP anti-contractile effect was completely reversed by NOS inhibitors showing CNP could activate NOS and NO production could be responsible for CNP anti-contractile effect in aortas. In the same way, Murthy et al. (1998) had demonstrated that a selective agonist of NPR-C, called cANP(4-23), can increase NOS activity via Gi protein and calcium influx. Costa et al. (2007) reported that CNP increased NOS activity in aorta homogenate and our research group (Andrade et al., 2014) has described endothelium-dependent relaxation induced by CNP in rat aortas that seems to lead to NPR-C activation, but with no alteration in phosphorylation status of Ser^1177^ of NOS3. As described by Anand-Srivastava et al. (1996), the activation of cytosolic domain of NPR-C leads to adenylyl cyclase inhibition via *Pertussis* toxin sensitive-G protein. Furthermore, Anand-Srivastava (2005) reviewed NPR-C intracellular signaling with inositol-trisphosphate (IP_3_) and diacylglycerol (DAG) formation, calcium mobilization, potassium channel, NOS, and PKC activation.

Although Nox1 protein expression was higher in CLP aortas, the basal activity of NADPH-dependent ROS production was low in CLP aortas, suggesting another source of ROS in CLP aortas. Moreover, Brandes et al. (1999) determined after LPS stimulus, that there are increased NADPH oxidase and xanthine oxidase expression in rat aortas, leading to ${\text{O}}_{2}^{–}$, H_2_O_2_ production and after reaction with NO to form peroxynitrite (ONOO^−^). Since NADPH oxidase and xanthine oxidase produce ${\text{O}}_{2}^{–}$ and H_2_O_2_, these results suggest the contribution of ${\text{O}}_{2}^{–}$ to the contractile effect to PE in Sham and CLP rat aortas. However, when intracellular H_2_O_2_ was removed, there were no changes on PE-induced contraction in both groups. It is possible that ROS production in VSM cells could compensate the removal of endothelial ROS on PE-induced contraction.

NADPH-dependent ROS production was increased after PE alone or CNP and PE stimuli, suggesting that CNP can positively modulate ROS production in rat aortas. In relation to the CNP anti-contractile effect in rat aortas, it seems dependent on H_2_O_2_, possibly through ${\text{O}}_{2}^{–}$ dismutation by SOD. As presented by Anand-Srivastava (2005), CNP could stimulate PKC which in turn activates NADPH oxidase (Brandes et al., 2014). Thus, NADPH-dependent ROS production increased by CNP and PE could be due to NPR-C activation dependent on PKC. Jao et al. (2001) reported low protein expression of PKCα in rat model of sepsis. The reduced effect of PE or CNP and PE on NADPH-dependent ROS production could be due to reduced PKC expression and/or PKC deficient activity in CLP aortas. In fact, PKCα mRNA expression was lower in CLP aortas compared to Sham. Furthermore, protein expression of NPR-C on VSM cells from CLP aortas was lower compared to Sham. It might suggest a reduction on protein expression or activity of the NPR-C-PKCα-NADPH oxidase axis in CLP aortas.

Weber et al. (1991) identified Anantin as the first competitive antagonist of NPR associated with cGMP formation, i.e., NPR-A and NPR-B receptors. Then, Anantin has been used as non-selective NPR-A/B antagonist. As CNP is the endogenous agonist of NPR-B (Koller and Goeddel, 1992) and has low affinity for NPR-A, Anantin could be considered a NPR-B antagonist for CNP effects. We demonstrated that CNP anti-contractile effect was dependent on NPR-B activation, since Anantin reversed this effect. Madhani et al. (2003) showed that CNP relaxation was inhibited by NPR-B antagonist in mice aortas. Rautureau et al. (2010) reported that CNP increased cGMP production independently of sGC in endothelial cells that was accompanied by NO production. Simon et al. (2009) showed CNP hyperpolarizes the endothelial cell membrane in a cGMP-dependent protein kinase (GK) manner. Activation of NPR-B by CNP was evident in Sham and CLP aortas. However, basal NPR-B activity without exogenous CNP was enough to modulate PE-induced vasoconstriction. Anantin at low concentration was able to increase PE maximum contraction only in CLP aortas. The greater effect of Anantin on high concentration suggests an important contribution of endogenous CNP for regulation of vascular tone. Besides, protein expression of NPR-B was not altered in aortas from Sham and CLP rats.

According to Hinder et al. (1997) and Stubbe et al. (2004) described that NPR-A/B antagonist preventing hypotension due to sepsis. This is the first mention that endogenous CNP or NPR-B intrinsic activity can contribute to modulation of vascular tone in aortas from Sham and CLP rats. Moreover, endogenous CNP production can be stimulated by several factors, such as LPS, TNF-α, IL-1α, and IL-1β, acetylcholine and bradykinin (Suga et al., 1993; Anand-Srivastava, 2005). Sepsis is a condition in which plasma levels of CNP are high (Hama et al., 1994). We demonstrated that endogenous CNP on vascular endothelium was lower in CLP than in Sham aortas. However, the peptide expression on VSM cells was greater in CLP than Sham aortas. It suggests a possible release of CNP from the endothelium to VSM in CLP aortas. Although the protein expression of NPR-B was similar in both groups, NPR-B activity might be different in CLP aortas.

Our results showed for the first time the CNP induced anti-contractile effect on PE-induced contraction that was greater in CLP than Sham aortas. This effect is dependent on NOS activity, ROS production and NPR-B activation. ${\text{O}}_{2}^{–}$ and H_2_O_2_ production seems to be part of the same signaling pathway, and PE alone or CNP and PE increase ROS production. In CLP aortas, CNP could be released from the endothelial to VSM cells. NPR-B intrinsic activity or endogenous CNP could modulate the vascular tone. Furthermore, the lower protein expression of NPR-C on VSM cells of CLP aortas and lower relative PKCα mRNA expression could contribute to the reduction of CNP-induced NADPH-dependent ROS production in CLP aortas. If the activation of NOS and NPR-B belongs to a common intracellular signaling remains to be investigated. Moreover, it is evident the contribution of CNP system on vascular hyporeactivity to α_1_-adrenergic activation in CLP aorta, and it might represents a potential target for future pharmacological studies.

## Author contributions

Conception of the work: LP, BRS, LMB. Design of the work: LP, AFP, BRS, AA, LCP, JETS, LMB. Acquisition and analysis of data: LP, AFP, BRS, AA, LCP. Interpretation of data: LP, AFP, BRS, AA, LCP, JETS, LMB. Drafting or revising of the work: LP, AFP, BRS, AA, LCP, JETS, LMB. Final approval: LP, AFP, BRS, AA, LCP, JETS, LMB. Agreement to be accountable for all aspects of the work: LP, AFP, BRS, AA, LCP, JETS, LMB.

## Funding

This study was supported by the Fundação de Amparo a Pesquisa do Estado de São Paulo and Conselho Nacional de Desenvolvimento Científico e Tecnológico.

### Conflict of interest statement

The authors declare that the research was conducted in the absence of any commercial or financial relationships that could be construed as a potential conflict of interest.

## Abbreviations

Ana: Anantin
AngII: Angiotensin II
ANP: Atrial natriuretic peptide
AUC: Area under the curve
BNP: Brain natriuretic peptide
cGMP: Cyclic guanosine-monophosphate
CLP: Cecal ligation and puncture
CNP: C-type natriuretic peptide
CO: Control
DAG: Diacylglycerol
DPI: Diphenylene iodonium chloride
DTPA: Diethylene triaminepentaacetic acid
FI: Fluorescence intensity
FITC: Fluorescein isothiocyanate
g.g^−1^: Gram of contraction per gram of dry tissue weight
GAPDH: glyceraldehyde-3-phosphate dehydrogenase
GK: cGMP-dependent kinase
H_2_O_2_: Hydrogen peroxide
HRP: Horseradish peroxidase
HUVEC: Human umbilical vein endothelial cells
IL: Interleukin
IP_3_: Inositol-trisphosphate
JNK: c-Jun N-terminal kinases
L-NAME: N^ω^-nitro-L-arginine methyl ester hydrochloride
LPS: Lipopolysaccharide
MAP: Mean arterial pressure
ME: Maximum effect
mmHg: Milimeters of mercury
mN.mm^−1^: Milinewton per milimeter
NADPH: Nicotinamide adenine dinucleotide phosphate
NaOH: Sodium hydroxide
NEM: *N*-ethylmaleimide
NO: Nitric oxide
NOS: Nitric oxide synthase
NOx: nitrite + nitrate
Nox1: NADPH oxidase 1
NPR: Natriuretic peptide receptor
${\text{O}}_{2}^{–}$: Superoxide anion
ONOO^−^: Peroxynitrite
PE: Phenylephrine
PEG: Polyethylene glycol
PKC: Protein kinase C
PLC: Phospholipase C
qPCR: Quantitative polymerase chain reaction
RLU: Relative luminescence unit
RMA: Resistance mesenteric arteries
ROS: Reactive oxygen species
Sham: Sham-operated rats
SOD: Superoxide dismutase
pGC: Particulate guanylyl cyclase
sGC: Soluble guanylyl cyclase
TNF: Tumor necrosis factor
U: Units
VSM: Vascular smooth muscle.
