# Supplementary material for: C-Type Natriuretic Peptide Induces Anti-contractile Effect Dependent on Nitric Oxide, Oxidative Stress, and NPR-B Activation in Sepsis
Source: Front Physiol. 2016 Jun 23;7:226. doi: 10.3389/fphys.2016.00226 (PMC4917550; doi:10.3389/fphys.2016.00226)
Supplement: Supplementary file 1 [file DataSheet1.docx]

Supplementary Data

**C-type natriuretic peptide induces nitric oxide, oxidative stress and NPR-B activation-dependent anti-contractile effect in sepsis**

Laena Pernomian, PhD^1^, Alejandro Ferraz do Prado, PhD^1^, Bruno Rodrigues Silva, PhD^2^, Aline de Azevedo, PhD^3^, Lucas César Pinheiro, PhD^1^, José Eduardo Tanus-Santos, PhD^1^, Lusiane Maria Bendhack, PhD^2,*^

**1 Detailed Methods**

**1.1 Animals**

##### All the procedures were performed in accordance with the standards and policies of the Ethics Committee on Animal Care and Use of the University of São Paulo (#144/2011). One hundred and thirty two male *Wistar* rats (200 g) were anesthetized with Tribromoethanol (0.25 g.Kg^-1^, i.p.); after the animals were undergone to cathetherization surgery on femoral artery to the measure of MAP. Animals were maintained on standard rat chow and water on their cases for 24h to complete recovery, as described previously (Araujo *et al*., 2011). On the next day, rats MAP were measured for basal values (before surgery), using pressure transducers (Disposable BP Transducer, model MLT0699 ,ADInstruments^®^). After, rats were anesthetized with Tribromoethanol (0.25 g.Kg^-1^, i.p.) and randomly submitted to cecal ligation and puncture (CLP) surgery to induce sepsis, with 12 punctures using a 16 gauge-needle on anti-mesenteric border of the cecum (Wichterman*et al.*, 1980, Fernandes*et al.*, 2009, Araújo*et al.*, 2011), or they were submitted to a medial laparotomy only (control-operated rats or Sham). Survival and MAP were analysed for 24h after surgeries. Animals were maintained on standard rat chow and water on their cases.

**1.2 Functional studies by vascular reactivity**

Sham and CLP rats were killed by decapitation under anaesthesia (inhaled Isoflurane),4h after the surgeries, and thoracic aorta and resistance mesenteric artery (RMA) were used to the measure of isometric tension. The thoracic aorta and gut were quickly removed and cleaned of adherent tissues. The gut was maintained on cold Krebs solution for small arteries until the isolation of RMA.

**1.2.1 Vascular reactivity studies on thoracic aorta**

The thoracic aorta was cut into rings (4 mm length) and placed between two stainless-steel stirrups and connected to an isometric force transducer (Panlab force transducers, model TRI201), to measure tension in the vessels. The values of contraction to PE (*y* axis on graphs relative to cumulative concentration-effect curves) were presented as grams of isometric tension divided by dry tissue of each aortic ring (g.g^-1^) to avoid any differences between the morphological changes in CLP aortas. However, dry weights were not different between the groups.Rings were placed in the organ bath chamber containing Krebs solution with the following composition (mmol.L^-1^): 130.0 NaCl, 4.7 KCl, 1.2 KH_2_PO_4_, 1.2 MgSO_4_.7H_2_O, 14.9 NaHCO_3_, 5.5 glucose, and 1.6 CaCl_2_. The solution was maintained at pH 7.4, gassed with 95% O_2_ and 5% CO_2_ at 37°C. The rings were initially stretched to a basal tension of 1.5 g; then, they were allowed to equilibrate for 60 min. Viability of aortas were evaluated by contraction induced by potassium chloride solution (60 mmol.L^-1^). Endothelial integrity was qualitatively assessed by the degree of relaxation induced by Acetylcholine (1 µmol.L^-1^) in the presence of contractile tone induced by Phenylephrine (PE, 0.1 µmol.L^-1^). The rings were discarded if relaxation induced by Acetylcholine was not 80% or greater. Cumulativeconcentration-effect curves were constructed to PE (0.1 nmol.L^-1^ to 10 µmol.L^-1^) in intact endothelium rat aortas. These curves were obtained in the absence (CO) of or after 30 min incubation with C-type natriuretic peptide (CNP, 10 nmol.L^-1^), N^ω^-nitro-L-arginine methyl ester hydrochloride (L-NAME, 100 µmol.L^-1^), N^ω^-propil-L-arginine (50 nmol.L^-1^), 1400W (10 nmol.L^-1^), Tiron (100 µmol.L^-1^), polyethylene glycol-Catalase (PEG-Catalase, 250 U.mL^-1^), Anantin (Ana, 0.1 µmol.L-1 or 1 µmol.L^-1^) alone or in the combination of each inhibitor/antagonist and CNP. The PE potency (*p*D_2_) and maximum effect in inducing contraction were evaluated.

**1.2.2 Vascular reactivity on RMA**

The gut was placed on a Petri dish and the RMA were isolated and cut into ring (2 mm length) using a dissection magnifier (Nikon, SMZ 645, USA) in cold Krebs solution for small arteries with the following composition (mmol.L^-1^): 119.0 NaCl, 4.7 KCl, 1.2 KH_2_PO_4_, 2.5 CaCl_2_, 1.2 MgSO_4_.7H_2_O, 25 NaHCO_3_, and 11.0 glucose. After the vessel isolation, the cold solution was replaced for 5 mL of Krebs solution maintained at pH 7.4, gassed with 95% O_2_ and 5% CO_2_ at 37°C. A tungsten wire (40 µm) was passed into the vessel and tied in myograph to resistance vessels (Danish Myo Tech, Multi Wire Myograph System 620M model, JP-Trading I/S, Aarhus, Denmark) and another one was connected in an isometric force transducer (PowerLab 4/26, ADinstruments^®^, Australia). The resistance mesenteric artery was allowed to equilibrate for 20 min and after this equilibration period the artery was stretched to an optimal resting tension regarding to its inner diameter, corresponding to the internal circumference related to a 100 mmHg transmural pressure to an *in situ* relaxed vessel (L100). The internal circumference was L1, calculated by the equation L1=0.90xL100, which the developed force is maximum. The luminal diameter was determined according to the equation I=L.π^-1^, using a resistance artery normalization software (DMT Normalization Module, ADInstruments^®^, Australia). Only the second branch of RMA with 200 µm to 350 µm of internal circumference were used. After the normalization procedures, the arteries were contracted with potassium chloride solution (120 mmol.L^-1^) to evaluate their viability. Endothelial integrity was analysed by the relaxation induced by Acetylcholine (10 µmol.L^-1^) in the presence of contractile tone induced by PE (10 µmol.L^-1^). The rings were discarded if relaxation induced by Acetylcholine was not 80% or greater. Cumulativeconcentration-effect curves were constructed for PE (0.1 nmol.L^-1^ to 100 µmol.L^-1^) in intact endothelium resistance mesenteric artery in the absence of or in the presence of CNP (10 nmol.L^-1^, 30 min). The area under the curve (AUC) of PE contraction was evaluated. The values of contraction to PE (*y* axis on graphs relative to cumulative concentration-effect curves) were presented as miliNewton of tension divided by the length of each RMA ring (mN.mm^-1^) to avoid any differences between contractions to PE in RMA. However, the length of these vessels was not different between the groups.

**1.3 Measurement of plasma NO metabolites**

Cardiac blood samples were collected from Sham or CLP rats 4h after the surgery in heparin-containing tubes with diethylene triaminepentaacetic acid (DTPA; 0.1 mmol.L^-1^) and *N*-ethylmaleimide (NEM; 8 mmol.L^-1^). Samples were centrifuged at 200 xg for 5 min. Plasma samples were collected and frozen at -70°C.

**1.3.1 Measurement of plasma nitrite concentration**

Plasma aliquots of Sham or CLP rats were analysed for their nitrite content using an ozone-based reductive chemiluminescence assay (Pinheiro*et al.*, 2012). Briefly, to measure nitrite concentration in plasma, 50 mL of plasma samples was injected into a solution of acidified triiodide, purging with nitrogen in line with a gas-phase chemiluminescence NO analyzer (Sievers Model 280 NO analyzer; Boulder, CO, USA). Approximately, 8 mL of triiodide solution (2 g potassium iodide and 1.3 g iodine dissolved in 40 mL water with 140 mL acetic acid) was placed in the purge vessel into which plasma samples were injected. The triiodide solution reduces nitrites to NO gas, which is detected by the NO analyzer. The data were analysed using the software Origin Lab6.1.

**1.3.2 Measurement of plasma NOx (nitrate + nitrite) concentration**

The plasma NOx concentration of Sham or CLP rats wasdetermined induplicate by using the Griess reaction (Pinheiro*et al.*, 2012). Briefly, 40 mL of plasma was incubated with the same volume of nitrate reductase buffer (0.1 mol.L^-1^ potassium phosphate, pH 7.5, containing 1 mmol.L^-1^ β-nicotinamide adenine dinucleotide phosphate and 2 U of nitrate reductase.mL^-1^) in individual wells of a 96-well plate. Samples were allowed to incubate overnight at 37°C in the dark. Eighty microliters of freshly prepared Griess reagent (1%sulfanilamide, 1%naphthylethylenediaminedihydrochloride in 5% phosphoric acid) was added to each well and the plate was incubated for an additional 15 min at room temperature. A standard nitrate curve was obtained by incubating sodium nitrate (0.2 mmol.L^-1^to 200 mmol.L^-1^) with the same reductase buffer.

**1.4 Western blotting analysis**

Thoracic aorta with intact endothelium isolated from Sham or CLP rats were frozen in liquid nitrogen and homogenized in RIPA buffer. Protein concentrations in the samples were determined by Bradford method. Corresponding volume of these homogenates of 30 µg protein were added with the same volume of buffer containing SDS (Tris-HCl 100 mmol.L^-1^, SDS 4%, bromophenol blue 0.1%, glycerol 20%), to negatively charge proteins to electrophoresis. Proteins of the samples were separated on 12% SDS-PAGE and transferred to a nitrocellulose membrane (GE Healthcare). Membranes were blocked for 60 min with 5% non-fat milk at room temperature. Then, membranes were incubated with mouse primary antibody anti-NOS2 (1:2500), or anti-NOS3 (1:2500), and rabbit primary antibody anti-NPR-B (1:5000) or anti-Nox1 (1:2000), overnight at 4°C. After that, membranes were incubated with a HRP-conjugated goat anti-rabbit (1:5000) or goat anti-mouse secondary antibody (1:5000) for 60 min at room temperature. Protein bands were visualized by means of chemiluminescence (ECL Millipore). Protein expression levels were normalized by mouse anti-β-actin (1:2000). Band intensities were quantified by densitometry using ImageJ Software (1.46r, Wayne Rasband, NIH, USA).

**1.5 Confocal microscopy analysis**

Thoracic aorta was isolated from Sham or CLP rats 4h after the surgeries, cleaned and immediately frozen on cryoprotection liquid (Tissue Tek – OCT, Sakura). Sheets were prepared with 10 µm of thickness and immunofluorescence was performed. Briefly, sheets were fixed with paraformaldehyde 4% for 15 min and incubated with bovine albumin serum 1% + goat normal serum 10% + glycine 0.3 mol.L^-1^ solution and PBS-Tween 0.1% for 1h. After, the primary antibodies were applied: goat anti-CNP (1:100, Santa Cruz) or rabbit anti-NPR-C (1:800, Abcam) and mouse anti-smooth muscle α actin FITC (1:100, Abcam) for 12h at 4°C. Following this, the secondary antibody mouse anti-goat Alexafluor 647 (1:1000, BD Pharmingen^TM^) or sheep anti-rabbit Alexafluor 647 (1:1000, GE Healthcare) was incubated for 1h, at room temperature. Fluoroshield^®^ with DAPI (Sigma Aldrich) was applied to stain nuclei at 4°C overnight. Sheets were sealed with formaldehyde-free colourless varnish at 4°C until complete drying. Images were acquired using confocal microscopy (Leica TSC SP5; Confocal Microscopy Laboratory – LMMC, FAPESP #2004/08868-0), with 63x oil objective. The Leica Application Suite – Advanced Fluorescence Lite 2.3.0 software (Leica Microsystems, 1997-2010) was used to capture images on TCS SP5 xyz mode, with 200Hz velocity and 1024x1024 format. Images were analysed using ImageJ software (1.46r, Wayne Rasband, NIH, USA) as total endothelium area, 20 areas of 100x50 for the vascular smooth muscle (VSM) layer, and 8 areas of 100x50 for adventitia layer.

**1.6 Lucigenin chemiluminescence analysis**

Vascular nicotinamide adenine dinucleotide phosphate (NADPH)-dependent ROS production was assessed in aortic ring with intact endothelium isolated from Sham or CLP rats on vascular reactivity, previously stimulated or not (basal) with PE (0.1 µmol.L^-1^) in the absence of or presence of CNP (10 nmol.L^-1^, 30 min). After that, the aortic rings were collected and immediately frozen on liquid nitrogen. At the moment of experiments, the frozen aortic rings were transferred to luminescence tubes with 1 mL of Hanks solution (pH 7.4) and lucigenin (5 µmol.L^-1^). After signal stabilization, NADPH (300 µmol.L^-1^) was added and the luminescence was continuously measured using Single Tube Luminometer Berthold FB12, at 37°C. Signals derived from aortic ring were subtracted from signals after the NADPH addition and the results were normalized by aortic rings dry tissue weight. Diphenyleneiodonium chloride (DPI, 10 µmol.L^-1^, 30 min), the non-selective flavoproteins inhibitor, was used as negative control of basal NADPH-dependent ROS production. Data were presented as relative luminescence units (RLU).mg^-1^.min^-1^. FB Sirius Software was used to data acquisition.

**1.7Quantitative polymerase chain reaction (qPCR) analysis**

### Total RNA was extracted from aortas homogenates isolated from Sham and CLP rats, with TRIzol^®^ reagent (Life Technologies, CA, USA) according to manufacturer’s instruction. RNA concentration was determined by measuring absorbance at A260 and A280 and the quality was assessed by gel electrophoresis. The cDNA was synthesized using 1 µg of RNA in a total volume of 20 μL using anchored randomhexadeoxynucleotidein the reaction conditions for transcriptor First-strand cDNA Synthesis Kit (GE Healthcare UK Limited, UK). The reaction was incubated at 65ºC for 10 minutes, stopped on ice and then incubated at 37°C for 1 hour.

### Quantitative RT-PCR were measured on an ABI Prism 7500 Sequence Detector system with SDS 2.1 Software (Applied Biosystems 7500 Real-Time PCR, Applied Biosystems, CA, USA), using SYBR^®^ FAST qPCR kit Master Mix Universal (Kapa Biosystems, Boston, USA) in a total volume of 20 µL, with 50 nmol.L^-1^ of primers for PKCα (protein kinase C α) (Zhao *et al.*, 2015) or GAPDH (glyceraldehyde-3-phosphate dehydrogenase) (Woodard *et al.*, 2002) (Integrated DNA Technologies, USA) and cDNA diluted to 1:100. The PCR cycles were proceeded as follows: 2 minutes at 50ºC, 10 minutes at 95ºC followed by 40 cycles at 95ºC for 15 seconds and 60ºC for 1minute.

To evaluate PKCα levels two replicate analysis were performed and the amount of target RNA was normalized with respect to the endogenous control (housekeeping) gene, GAPDH. Data were expressed according to the 2^∆∆Ct^ method using the mean value of the ∆Ct of the control group as the calibrator (Sham). The amplification efficiency for each primer pair was evaluated at a 1:10 serial dilution and was adequate for qPCR analysis. Data regarding primer characteristics, amplification efficiency, and product length and guanosine-cytosine content are presented on Table 1. The primers amplification efficiencies were calculated using “10^(-1/slope)-1^ x 100”.

**1.8 Drugs and solutions**

Phenylephrine, Acethylcholine, L-NAME, 1400W, PEG-catalase, Tribromoethanol, PBS, DPI, lucigenin, NADPH, NEM, DTPA, primary antibody mouse anti-β-actin, mouse anti-Nox1, and Fluoroshield^®^ with DAPI were purchased from Sigma Aldrich. CNP was purchased from American Peptide Company. Anantin was purchased from Bachem. Tiron was purchased from Riedel-deHaën. N^ω^-propil-L-arginine was purchased from Tocris. Isoflurane was purchased from Abbott. Primary antibodies rabbit anti-NPR-B,rabbit anti-NPR-C, andanti-smooth muscle α actin were purchased from Abcam. Primary antibodies mouse anti-NOS2, mouse anti-NOS3, and secondary antibody mouse anti-goat Alexafluor 647 were purchased from BD Pharmingen^TM^. Primary antibody goat anti-CNP was purchased from Santa Cruz. Secondary antibodies HRP-goat anti rabbit, HRP-rabbit anti mouse and goat anti-rabbit Alexafluor 647, and First-strand cDNA synthesis kit were purchased from GE Healthcare.SYBR^®^ Green PCR Master Mix was purchased from Kapa Biosystems.TRIzol**^®^** was purchased from Life Technologies. PKCα and GAPDH primers were purchased from Integrated DNA Technologies.

Phenylephrine, Acethylcholine, L-NAME, 1400W, N^ω^-propil-L-arginina, PEG-catalase, DPI, PBS, and Tiron were diluted in miliQ water. CNP was diluted in 5% acetic acid and after in miliQ water. Lucigenin was diluted in 10 mg.mL^-1^ acetic acid solution. NADPH was diluted in 0.01 N NaOH (50 mg.mL^-1^) solution. Anantin was diluted in 50 mmol.L^-1^ acetic acid solution. Primary and secondary antibodies were used as manufacturing specifications. Krebs solution was prepared in miliQ water.

**1.9 Statistical analysis**

Results are presented as the mean ± S.E.M. Each experimental *n* represents samples isolated from different animals. Comparisons between groups were conducted by the Student *t* test, One-way analysis of variance followed by Newman-Keuls, or Two-way analysis of variance using Bonferroni correction for multiple comparisons, as appropriate. The level of statistical significance was defined as *P*<0.05.

**2 Supplemental Table**

Table 1: PKCα or GAPDH primers characteristics used for qPCR analysis. The primers amplification efficiencies were calculated using “10^(-1/slope)-1^ x 100”.

| Characteristics | Primers | |
| --- | --- | --- |
|  | PKC α (Zhao *et al.*, 2015) | GAPDH (Woodard *et al.*, 2002) |
| Sense | 5’- CGGATAAGGGACCTGACACT-3’ | 5’-TTCCAGTATGACTCTACCC-3’ |
| Antisense | 5’- ACGCACTGCTTGTGAACATT-3’ | 5’-ATGGACTGTGGTCATGAGCCC-3’ |
| Slope | -3.34 | -3.79 |
| Amplification efficiency | 99.3% | 83.0% |
| Product length (bp) | 166 | 399 |
| Guanosine-Cytosine content: sense | 55.0% | 47.4% |
| Guanosine-Cytosine content: antisense | 45.0% | 57.1% |

bp: base pairs.

**3 Supplemental References**

Araújo, A.V., Ferezin, C.V., Rodrigues, G.J., Lunardi, C.N., Vercesi, J.A., Grando, M.D. et al. (2011). Prostacyclin, not only nitric oxide, is a mediator of the vasorelaxation induced by acetylcholine in aortas from rats submitted to cecal ligation and perforation (CLP). *Vasc.Pharmacol*.54(1-2), 44-51.

# Fernandes, D., Sordi, R., Pacheco, L.K., Nardi, G.M., Heckert, B.T., Villela, C.G. et al. (2009). Late, but not early, inhibition of soluble guanylate cyclase decreases mortality in a rat sepsis model. *J. Pharmacol. Exp.Ther*. 328(3), 991-999.

Pinheiro, L.C., Montenegro, M.F., Amaral, J.H., Ferreira, G.C., Oliveira, A.M., Tanus-Santos, J.E. (2012).Increase in gastric pH reduces hypotensive effect of oral sodium nitrite in rats. *Free Rad. Biol. Med*.53, 701-709.

Wichterman, K.A., Baue, A.E., Chaudry, I.H. (1980). Sepsis and septic shock – a review of laboratory models and a proposal. *J. Surg. Res*. 29(2), 189-201.

Woodard, G.E., Rosado, J.A., Brown, J. (2002). Expression and control of C-type natriuretic peptide in rat vascular smooth muscle cells. *Am. J. Physiol. Regulatory Integrative Comp. Physiol*.282, R156-R165.

Zhao, Y., Vanhoutte, P.M., Leung, S.W.S. (2015). α_1_-Adrenoceptor activation of PKC-ε causes heterologous desensitization of thromboxane receptors in the aorta of spontaneously hypertensive rats. *Br. J.Pharmacol*.172(14), 3687-701.
